# Patient-centered perspectives: A qualitative evaluation of the Hip Instructional Prehabilitation Program for Enhanced Recovery (HIPPER)

**DOI:** 10.1371/journal.pone.0322114

**Published:** 2025-04-24

**Authors:** Aditya Dhariwal, Somayyeh Mohammadi, Ethan Simpson, Marie D. Westby, Wendy Watson, William C. Miller

**Affiliations:** 1 Department of Occupational Science and Occupational Therapy, University of British Columbia, Vancouver, BC, Canada; 2 Rehabilitation Research Program, GF Strong Rehabilitation Centre, Vancouver, BC, Canada; 3 Centre for Aging SMART, Vancouver Coastal Health Research Institute, Vancouver, BC, Canada; 4 Vancouver Coastal Health Research Institute, Vancouver, BC, Canada; 5 Vancouver Coastal Health, Vancouver, BC, Canada; IRCCS: IRCCS Ospedale San Raffaele, ITALY

## Abstract

Over 55,000 total hip replacement (THR) surgeries were performed in Canada in 2021, with the number rising each year. Excluding rehabilitation, the cost of hip replacement procedures exceeded $675 million, a large burden on the Canadian healthcare system. Considering this large financial impact, prehabilitation delivered through electronic health (eHealth) can improve post-surgical outcomes and reduce overall healthcare expenditures by enhancing recovery and reducing hospital length of stay. This study utilized grounded theory to examine user experience of the Hip Instructional Prehabilitation Program for Enhanced Recovery (HIPPER), an eHealth approach to prehabilitation education. Participants were purposively sampled and conventional content analysis was conducted on 18 transcribed semi-structured interviews with participants who completed the HIPPER program in preparation for THR surgery. We identified three categories and seven subcategories: 1) ‘That wasn’t so hard!’, with the subcategories *easy to use*, *learning information* and *appreciating HIPPER*, which describe how participants were able to learn information and use the modules easily; 2) ‘I’m ready’, comprising of the subcategories *being prepared for* surgery and *having a smooth and good* experience which relates to how participants were prepared and confident going into surgery; 3) ‘I’d like to have seen’, including the subcategories *having differing experiences* and *suggesting* additions, which explore participants’ constructive criticism and ideas for improvement. Our results show useful features to include in eHealth programs and demonstrate how educational prehabilitation in the form of eHealth is helpful and usable for older adults undergoing THR. We also discuss and inform the integration of feedback and development of eHealth programs for elective surgical procedures. This study was registered with ClinicalTrials.gov on November 21, 2016 with the registration number NCT02969512.

## Introduction

In Canada, approximately 26.6% of individuals aged 50 and older have physician-diagnosed osteoarthritis (OA), which can break down joint cartilage and lead to joint pain, reducing mobility [1]. When individuals do not respond to first-line approaches including education, exercise, weight loss and medications, total joint replacement surgery becomes an option [2]. Total hip replacements (THRs) and total knee replacements (TKRs) manage pain and improve mobility, increasing the quality of life for patients [3]. THR was the second most performed inpatient surgery in Canada in 2020–2021 [4] with over 55,000 surgeries performed [5]. While this number was lower than previous years due to the COVID-19 pandemic [6], the number of THRs in Canada over the past 20 years have been increasing steadily, at a rate of 5.0% over the past three years [5]. Accompanying this steady increase in volume is an increasing burden on Canada’s healthcare system.

In 2020–2021, the annual estimated inpatient costs for hip replacements in Canada exceeded $675 million, excluding costs associated with rehabilitation, travel, lodging, education and equipment [5]. While specific figures including rehabilitation costs are unavailable in this estimate, the total economic burden is likely much higher when considering post discharge care. Prehabilitation, a term used to encompass pre-operative education and exercise, increases patients’ preparedness for surgery, reduces recovery time and improves other post-surgical outcomes, reducing costs related to hospital length of stay [7–9]. Education in prehabilitation varies on the program but can include information on nutrition, exercise, pain management and psychological support [10]. This education aims to manage patient expectations and reduce anxiety related to the surgery [11]. It also encourages and facilitates positive health behavior changes not only pre-operatively but also in the long term [10]. By equipping patients with necessary skills such as pain management and exercises to improve functional mobility, this reduces admission into acute rehabilitation [12], reducing associated costs and providing better surgical outcomes [7–12].

Traditionally, prehabilitation is delivered using a face-to-face format, however this mode of delivery has disadvantages for both the patient and the hospital, such as travel time for the patient and expenses for the hospital [13]. However, with the emergence of newer technologies such as electronic health (eHealth), delivering prehabilitation programs remotely has become more feasible. eHealth is defined as the provision of healthcare services to improve health and wellbeing through the use of technology [14]. Various digital technologies integrate eHealth tools and rehabilitation, including online modules, tele-rehabilitation platforms, mobile applications, and remote monitoring systems [15]. eHealth tools allow for personalized and interactive programs tailored to individual needs and progress. They enable healthcare providers to monitor adherence to protocols remotely, track progress such as exercise frequency and communicate when necessary [16]. Patients can access resources and programs from the convenience of their homes, mitigating the costs associated with travel, parking, and accommodation for those living out of town [17,18]. This is especially important for rural communities, older adults, and people with mobility impairments.

Research has evaluated the effect of prehabilitation programs on THR outcomes, with a systematic review identifying 14 articles and a literature review identifying nine articles on THR prehabilitation programs [9,19]. The systematic review found that prehabilitation significantly improved scores in pain tests, with intervention groups reporting significantly less post-surgical pain on the Visual Analogue Scale (p=0.04) [9]. The literature review found that 91% of participants were satisfied with virtual prehabilitation and felt more prepared for their surgery [19]. These results suggest that prehabilitation is an effective, feasible and preferable tool for THR patients. However, they also reveal limitations. For instance, while one review found prehabilitation exercise therapy to be an effective measure, they also found that prehabilitation in the form of education has no statistical effect on postoperative physical functioning [9]. Importantly, out of all the included articles, only two used eHealth to deliver prehabilitation. One evaluated a synchronous strengthening exercise session in Montréal, Canada [20], while the other tested an education program called My Hip Journey in Australia [21]. Both studies included participants aged 18 and older and none of the studies in the reviews included interviews or a qualitative evaluation. This gap is significant because older adults, who represent the majority of patients undergoing THR [5], may have unique perspectives and challenges that are not captured by quantitative methods alone. Thus, there is a need for research that incorporates qualitative methods to gain a more in-depth understanding of users’ experience and evaluate the acceptability of eHealth prehabilitation amongst older adults.

Based on stakeholder perspectives identified by Reid et al [22], we developed an eHealth tool called Hip Instructional Prehabilitation Program for Enhanced Recovery (HIPPER). HIPPER is an interactive, educational prehabilitation program that focuses on individuals undergoing THR. Our qualitative study aims to evaluate the experiences and perspectives of patients who used HIPPER to prepare for THR surgery. More specifically, we evaluate participants’ experiences about using an online, asynchronous program to prepare for surgery, the relevance and usefulness of the information conveyed, the user-friendliness of the program, and possible refinements to the tool.

## Methods

### Research design

In this qualitative study, we used Qualitative Descriptive Design as that can be used in health care settings and mixed-method studies to investigate participants' perception on the quality of the intervention [23]. This qualitative study was part of a larger two-year randomized controlled trial (RCT) examining the feasibility of HIPPER [24]. Detailed information about recruitment, participant timeline, data collection and monitoring, group allocation, and blinding can be found in the RCT protocol paper [24]. To prepare this manuscript, we used the Standard for Reporting Qualitative Research (SRQR) checklist [25]. The trial was prospectively registered in ClinicalTrials.gov NCT02969512.

### Participants

The inclusion criteria for participants were: aged 50 years or older with physician-diagnosed hip OA, have internet access, and be scheduled to for unilateral THR 12 or more weeks after the time of recruitment. A local OA prehabilitation preparation program recruited individuals through email invitations and phone calls. Recruitment started on 10^th^ March 2021 and ended on 16^th^ July 2021.

Our study was approved by the University of British Columbia Research Ethics Board (H16-02553) and the Vancouver Coastal Health Research Institute (V16-02553). All participants gave written informed consent to a research assistant prior to enrolment in the study.

A research assistant invited all 26 participants who used HIPPER and completed the study to participate in an in-depth individual, semi-structured interview 30 days post-surgery. Eighteen participants agreed to an interview. The 30–60-minute interviews were conducted using a University of British Columbia licensed Zoom account. A trained individual conducted the interviews, piloting an interview guide on n=1 individuals. We aimed to collect participants’ experiences and preferences of the modules, the online modality of delivery and suggestions for improvements. Interviews were recorded using the zoom recording feature and transcribed verbatim by a research assistant.

### HIPPER and its development

HIPPER is a prehabilitation program developed through feedback from clinicians, patients who had or were waiting for a THR and their caregivers [22]. The program consists of 12 comprehensive modules delivered asynchronously online: osteoarthritis and hip replacement surgery, exercise before surgery, preparing your home for surgery, check your knowledge – set one, pain management before surgery, general health before surgery, check your knowledge – set two, two weeks before surgery, at the hospital, after surgery, check your knowledge – set three, resources.

Each module includes learning objectives and uses plain language throughout. There are mixed delivery formats such as written text, videos, images, and narrated slides. Check your knowledge sections (quizzes) are also included to facilitate knowledge retention and keep the learning engaging. Some slides require interaction, such as clicking on different equipment in a picture of a house to learn more. Participants can complete the modules wherever they wish and at their own pace. When they stop a module, their progress is saved, and they can choose to start where they left off when they return.

### Analysis

We assigned each participant a study ID prior to the interview. Interview data was transcribed verbatim using the zoom transcription feature and imported into Microsoft Excel by a research assistant. Personal identifiers were removed from the transcripts. Two team members independently coded the transcripts following principles of conventional content analysis in that codes and categories came from the data [26].

Content analysis was chosen as it allows phenomena to be described directly from the data without preconceived theoretical frameworks. Given that HIPPER represents a novel eHealth prehabilitation program, content analysis provided a practical framework for organizing diverse responses while remaining grounded in the data. This approach allowed us to understand participants’ experiences and preferences in their own words.

After coding the same three transcripts, they met to group codes into a codebook, which was then reviewed by two other researchers. This step deviated from traditional conventional content analysis but was more practical, providing structure and improving the efficiency of coding. Using this codebook, the rest of the transcripts were coded separately. Through an iterative process, coders collaborated twice more to update the codebook with new codes which the research team reviewed and edited. Similar codes were grouped into subcategories and larger categories with feedback from the research team. We then assigned representative quotes to each subcategory.

### Trustworthiness strategies

We used investigator triangulation by having coders independently code and compare the initial three transcripts and then subsequently meet with the broader team to ensure consistency in coding [27]. The research team used reflexivity by considering how their background, knowledge and values influenced the creation of categories [28]. Three members of the research team (MW, SM, WCM) have PhDs. SM is a woman in her 30s and a registered clinical counsellor, WCM is man in his 60s and a registered occupational therapist and MW is a woman in her 60s and a registered physical therapist. ES is a man in his 20s, has an MSc in rehabilitation sciences and conducted the interviews. The coders (AD, Rita Jin) were undergraduate students pursuing a Bachelor of Science working as research assistants. They were trained in coding and received guidance from ES, SM, and WCM. All members of the research team were affiliated with the University of British Columbia at the time of the study.

## Results

### Demographics

Of the 18 participants who were interviewed, 11 participants were female, and all underwent primary total hip replacement surgery between 2021 and 2023. When asked about their gender, all participants identified consistent with their biologically assigned sex. The mean age was 63 ± 8. A total of 15 participants were from Canada, seven participants lived alone, and nine participants were employed. The sample was well educated with 16 participants having attended higher education. Additionally, 17 participants self-reported good, very good, or excellent general health status.

### Categories

Our team identified three main categories and seven subcategories. The categories are: 1) “That wasn’t so hard!”, comprising of the subcategories *easy to use, learning information* and *appreciating HIPPER.* 2) “I’m ready,” including subcategories *being prepared for surgery*, and *having a smooth and good experience*; 3) “I’d like to have seen,” comprising of the subcategories *having differing experiences* and *suggesting additions*.

#### Category 1: ‘That wasn’t so hard!’.

This category emphasized how easy HIPPER was to use and how the information was clear, relevant, and well explained. It also reflected how preparation for a major surgery is not as daunting as it seems.

*Easy to use* reflects the participants’ comments about the functionality of the HIPPER modules.

Many participants remarked generally having a hard time with technology; however, they still found HIPPER easy to navigate and use. “[I found working through the modules] simple, and I’m a person who generally has difficulty with technology” (man, 70 years). Furthermore, many participants appreciated how organized HIPPER was, making the preparation for surgery smooth.

“It pieced the information into really digestible chunks, and also the way the modules were organized was useful because it was a nice gradual progression towards the surgery date” (woman, 54 years).

While the content is presented in a logical and chronological order, participants were able to jump to different modules if they wanted, which made HIPPER customizable and therefore helpful to individual situations.

Most participants were quite happy with the online delivery and the many benefits that came with it, such as not having to travel to receive an in-person lecture, especially for participants living in more rural settings. Another benefit all participants appreciated was being able to revisit and relearn forgotten information.

“I think the option of breaking it up and the option of repeating it or if you do miss something or thinking [you] misunderstood something and going back is obviously preferable to just a one-time boom there it is” (man, 70 years).

The asynchronous aspect of HIPPER was especially helpful for participants who did not have a set date for surgery. Having continued access to HIPPER allowed them to learn at their convenience and to their preference.

Participants also noted that in the preparation before surgery, they received information from many different sources such as their friends’ experiences, equipment booklets from the Red Cross, and checklists from their surgeons, which made it hard to keep track of relevant information. Referring to HIPPER, one participant remarked, “You’re not looking for a bunch of different papers. It’s all there in a package for you” (woman, 59 years), appreciating having all the information easily accessible in the HIPPER program.

*Learning information* speaks to the participants’ reflections regarding the content included in the module.

All participants thought that they learned relevant and useful information from HIPPER. Most participants were happy with the clarity and level of detail of the content, remarking, “You know, it was just enough. And it was funny because it covered what you needed to know without creating any kind of nervousness, or any kind of questions” (woman, 54 years)

HIPPER utilizes different learning styles such as videos, text, quizzes and images. This catered to the variety of learning styles that participants had. As a result, all the participants were happy with the online modality of delivery.

“I like the mix. So, for me, where there was a narrator, giving the information first with the text to read, and then maybe a little video that reinforced what they were saying or demonstrated it like with those conversations with people. And then I liked the little quizzes at the end” (woman, 54 years).

While most participants were happy with the way HIPPER delivered information, there were diverging opinions for certain sections, such as patient interviews. Despite appreciation for patient interviews and testimonials, one participant found them self- explanatory and tedious. “I think the only part that I kind of got bored at were the interviews with people” (woman, 79 years). The participant thought that the motivation to exercise was self-motivated, and videos would not change that. Overall, the feeling was that HIPPER presented clear, relevant and detailed information with a variety of learning styles allowing users to learn easily at their own pace as they prepared for a major surgery.

*Appreciating HIPPER* demonstrates the participants’ gratitude for the HIPPER modules.

Participants appreciated receiving HIPPER and attributed their successful recovery to it.

“What you gave me was awesome. It helped me perfectly, you know, it’s amazing. [...] And you know it’s helped me through everything. It gave me a better life [...] I’m glad I was a part of it” (woman, 61 years).

Participants were asked how HIPPER compared to their expectations for it. Many participants had experiences contrary to their low expectations. They found HIPPER to be engaging and interesting, making it easy to complete the self-paced program. When asked about how engaging HIPPER was, a participant remarked,

“I thought it was spot on myself, it helped me understand everything that was going on and I was quite interested in it too, just to see what I was about to experience through it all” (woman, 61 years).

In addition, many participants expressed a desire for eHealth tools for different procedures to benefit themselves, friends, and family members. “I’m really glad I was able to be part of it. Yeah, yeah, I think it’s a really great idea. And it’s funny because I have a colonoscopy booked and I’m really hoping that they have something online now” (woman, 54 years). Participants were grateful and expressed positive attitudes towards HIPPER, with many participants not wanting to change anything.

#### Category 2: ‘I’m ready’.

This category demonstrated how HIPPER prepared the participants and their supporters for a major surgery. It explains what aspects of HIPPER prepared them for surgery and how it made post-surgery recovery easier.

*Being prepared for surgery and recovery* reflects the aspects of the HIPPER modules that participants found useful.

Almost all the participants were satisfied with the way HIPPER prepared them for surgery, expressing gratitude for specific modules. The exercise module was frequently highlighted as helpful as it explained the importance of going into the surgery in good shape. “The module about health before surgery, which I think is so important. I think it’s why, because I was healthy before surgery, why I had a really good experience” (woman, 65 years). Additionally, most participants emphasized the helpfulness and relatability of past patient interviews. They found these videos reassured and boosted their confidence going into the surgery.

Participants highly regarded the equipment module as it made the preparation for post-surgical care much easier. Participants gained confidence learning hip precautions, house preparation and useful tips. This module also provided direct resources to purchase necessary materials. “Being able to say, okay, this is what you’re supposed to do, check, [...] gives you a little bit of, well gives you self confidence that you’re ready. [...] They gave a peace of mind” (woman, 65 years).

Another module that participants appreciated was pain management. Many participants were experiencing pain and discomfort and appreciated learning strategies to reduce pain and what to expect. They were grateful for the emphasis put on this module as it directly correlated with being better equipped for surgery.

Other modules that participants enjoyed included nutrition and the surgical process. Participants remarked “the nutrition part was actually quite good, and knowing what has to be done before the surgery. Like, what pills we are not allowed to take, which a lot of the doctors don’t tell you” (woman, 59 years). In one case, the information about the process encouraged one participant to get the surgery.

From using HIPPER, most participants noted being able to get everything ready, understand the process and know what to do. “The key was probably what to expect before the surgery and after. [...] So, going through the module helped clarify a lot of the fears that I would have.” (woman, 59). This established and managed participant expectations, removing the daunting aspect of the unknown. HIPPER also helped to correct misconceptions and created a positive mind-set, thus reducing anxiety.

Overall, most participants had increased confidence and reassurance going into the surgery. Participants felt prepared and did not feel as if they had missed any important pieces of information.

*Having a good and smooth experience* speaks to the peace of mind HIPPER gave participants.

Even if participants had previous knowledge or understanding about preparation for THR, participants highlighted the emotional benefit of going through HIPPER, finding it soothing.

“For me it was more of an emotional thing about just laying it out very calmly and very reassuring. [...] It made me better prepared” (woman, 54).

A participant who had multiple surgeries prior to THR said, “I’ve had 34 surgeries so this was one of the easiest that was prepared for and set up to go when I got home” (woman, 61 years). In addition to useful content, HIPPER emphasized the importance of having a support system which allowed participants to manage their expectations and have support ready. By emphasizing the serious nature of surgery, while making preparation straightforward, HIPPER contributed to a smooth preparation, and subsequent recovery.

A benefit seen from HIPPER was patients being able to bring knowledge to their healthcare teams and ask them informed questions, increasing their confidence and making the process better for both the patient and healthcare providers. One participant described how the care team at the hospital was impressed with his preparedness. Overall HIPPER helped create a strong mindset and allowed users to have a good and smooth experience throughout their surgery and recovery.

#### Category 3: ‘I’d like to have seen’.

This category focused on potential changes and additions that can be implemented into HIPPER. It highlighted aspects of HIPPER that were not as helpful and identified areas of improvement.

*Having differing experiences* reflects on participants’ experiences that deviated from their expectations with HIPPER.

The majority of participants had a positive experience with HIPPER, attributing their success and smooth experience to the content and presentation of the program. However, some participants experienced technical difficulties or had diverging experiences compared to those presented in HIPPER. As a result, a few participants did not find HIPPER to be personalized (relevant to their situation).

One participant found the program slow as she felt the pace was more suited towards less computer literate seniors. “I just found it slow” (woman, 55 years). On the other side of computer literacy, a few participants experienced difficulty accessing and using HIPPER online. Sometimes participants lost access to HIPPER through technical issues, “I couldn’t get in” (woman, 79 years) and sometimes participants found logging in or navigating HIPPER difficult “it was complicated to me” (woman, 61 years). To combat this, more detailed log in instructions were suggested, “there would be an audience that could actually use a step-by-step guide” (woman, 55 years). Most people used a laptop, computer or iPad to complete the program, however one participant used a phone to access the program. They noted that the program was more user friendly for their laptop.

Experiences that differed from participants’ expectations were related to specific items of information in HIPPER rather than entire sections. In one case, the videos made putting on pants seem easier than it was. “They made it look so easy. It was not easy at all” (woman, 79 years). Another participant had a unique negative experience with the explanation of the anesthetic used in surgery, suggesting a better explanation. “That really freaked me out. I think it could have been explained better” (woman, 58 years).

Five participants did mention a few equipment suggestions being not applicable to their situation. This was based on personal preference. One participant mentioned the equipment purchase recommendations were not as relevant for people who were not living in major cities. “There’s a few little changes that when you’re in Yukon that you wouldn’t see in Vancouver or bigger centers” (man, 67 years). Despite this, they mentioned “you can’t do it individually that way” (man, 67 years), explaining how adding such a specialized slide would mean the module would have to be specifically tailored for one individual which would not be feasible on a larger scale.

Finally, six participants noted not using or revisiting HIPPER post-surgery. Instead, they used the pamphlets and exercise books provided by the hospital upon discharge. “I had no needs that I thought could be met by looking at modules post-surgery” (man, 69 years). However, they still credited HIPPER for their good post-surgery experience.

*Suggesting additions* demonstrate potential refinements identified by participants.

Participants suggested additional beneficial features. A few participants noted that it was hard to visually differentiate between completed modules and to-do modules. There were some recommendations for more colour contrast to differentiate the two. “That might be made more clear in terms of the contrast of you know the colour, like the little checkmark, or little boxes change” (man, 69). This would also benefit the participants who experienced technical glitches and were not able to resume where they left off last. Another suggestion was to have the checklist pinned or more visible on the main menu, “Having it more visible on the toolbar [...] rather than having the checklist kind of buried in the modules” (woman, 54).

There were few suggestions on the depth of the content. However, one participant wanted more explanation on why certain emphasised content, such as hip precautions, were important to learn. Building on the lack of use of HIPPER post-surgery, discussed in the *having differing experiences* subcategory, one participant mentioned the need for detailed explanations for post-surgery activity. While they were mostly referring to the book provided by the hospital, they did also mention a lack of explanation in HIPPER. “I actually think that the one thing that is missing really in both [the booklet and HIPPER] is a little bit more detail in terms of the post-surgical activity that is needed” (woman, 55). The participant emphasised the need to encourage patients to continue doing exercise even after the six-month post-surgery mark.

Participants also discussed specific tips such as utilising a golfer’s reach for grabbing items or reversing your crutch for getting into and out of bed. Other tips suggested were knowing your weight prior to the surgery so you can provide an accurate value to the anaesthetist, and bringing in your own food as participants did not enjoy the hospital food. Furthermore, there were some equipment suggestions. One participant found a foam wedge useful to sit on the bed post-surgery while another participant utilised a grabber. There were some suggestions such as utilising a grabber or additional resources for food services that were already present in HIPPER, however this could be attributed to the long time between HIPPER use and the interview.

Other suggestions centred around alternative workouts when participants struggled to complete the outlined exercises. One participant noted being in too much pain to complete the regular exercises and wished HIPPER had some form of cardio workouts. Instead, they used online deep-seated cardio workouts to stay fit while waiting for surgery. “I think if someone’s going to wait for a long long time for surgery, then I think that might be useful” (woman, 77). On the same note, another participant also found upper body workouts useful. “Doing the upper body exercises really made me feel better” (woman, 54).

The main suggestion made by participants was the desire for assistance. One option given was a communication portal which would allow them to ask questions. “The only thing that I found that I would have liked to have had [was] somebody to ask questions” (woman, 68). However, when all participants were asked if they felt like they wanted to ask questions while they were going through HIPPER, some participants said they did not feel the need to. A second option was to have a feedback survey after a few modules to ascertain how participants were finding the program and whether they needed any assistance. This way the team could determine whether someone was struggling or not. “Have a little click, a little survey monkey. How was that for you? And then a second one, do you need assistance or somebody to go over it with you?” (man, 56). The participant who suggested this noted that they saw many elderly patients in pain at the hospital and thought it would be helpful for the older demographic to receive assistance with HIPPER if needed.

## Discussion

Our findings illustrate the experiences and perspectives of individuals who used HIPPER to prepare for THR surgery and recovery, showing it to be a usable and effective prehabilitation tool.

eHealth has a broad definition, and a wide range of different program formats can fall under eHealth [14]. When designing programs, researchers can tailor the program length, communication type used, level of collaboration and many other factors. In choosing these factors, researchers must keep in mind elements such as the target population, program goals, stakeholder opinions and the advantages, disadvantages and sustainability of each of these factors [29,30]. In our study, participants highlighted asynchronous delivery as an important benefit. Due to the large number of Canadians undergoing THR annually, and one of the goals of eHealth being to reduce the burden placed on the healthcare system, an asynchronous format mitigates the issues of limited healthcare provider time and long waits for healthcare services. Asynchronous communication can deliver similarly effective information as synchronous communication with added flexibility [31]. The asynchronous format allowed participants to revisit modules, a feature participants valued, stating that it helped them relearn forgotten information and clarify uncertainties. This feature, in addition to providing prehabilitation early, allowed for material review and temporally spaced practice, which are linked to better long term knowledge retention and educational outcomes [32,33]. Being able to revisit information was especially valuable in this study as it was conducted during the peak of COVID-19, when surgery dates were frequently delayed. Thus, delivering prehabilitation programs asynchronously allows information to be learned at times best suited for patients, facilitates knowledge retention and reduces burden on healthcare providers.

Success and learner performance in asynchronous learning environments are often positively correlated with motivation, engagement and active learning communities [34–38]. HIPPER participants mentioned being highly motivated due to the serious nature of major surgery and the desire for positive outcomes. However, it is important to consider a potential volunteer bias, as all interviewees had completed the program. Thus, this self-motivation cannot be implied for all THR patients. Instead, it would be beneficial to examine ways to increase motivation and engagement when designing eHealth programs. Viable options include positive reinforcement and encouragement [35] as well as adding a form of interaction with other users, such as a discussion board [39]. Adding encouraging messages at the end of modules could motivate participants to continue learning, and a collaborative discussion board could give them the opportunity to engage further with the material and their peers. It will be important to consider these additional features to increase motivation and engagement, thereby deepening patient understanding of the key concepts presented in prehabilitation education.

Delivering programs online has benefits such as a consolidated package of information and easy access. Participants reported receiving information from many different sources such as the Red Cross, their surgeon and a local prehabilitation group’s pamphlets, and appreciated not having to keep track of different papers and packets with HIPPER. However, the majority of THR patients are older adults [5], thus it is important to make sure programs are usable and acceptable to this demographic, as it is associated with decreased technological skill [40]. However, the literature shows an increase in technological use by older adults over the last few years [41]. In British Columbia (BC), 87.9% of adults 45–64 years and 56.0% of adults 65 years and older use the internet [42], supporting the use of eHealth programs in this demographic. Participants mostly expressed positive opinions navigating through the program and using an online service for prehabilitation, demonstrating the ability of older adults to use eHealth tools. Overall, the benefits and positivity reported by participants speak to HIPPER’s learnability, efficiency, memorability, satisfaction and few errors, which Souza and Lopez [43] identified as criteria for usability of eHealth programs. Keeping this criterion in mind when developing eHealth programs can be useful to ensure its effectiveness. Despite finding the platform user-friendly, a few users experienced difficulty logging in to the program, had technical difficulties returning to the correct module and suggested more contrast between completed modules and to-do modules. Providing more detailed instructions with images for logging in and adding contrast are potential additions that would mitigate such technical difficulties in the future and further enhance the usability of eHealth for older adults.

Participants offered suggestions according to individual preferences, including different equipment and specific tips. The main suggestion echoed by participants was the desire for a communication portal or someone to direct questions to. However, there were still varying opinions about this as some participants said they did not have any desire to speak to anyone or seek clarification at any point during the program. Another eHealth program *My Hip Journey* contained a feature allowing participants to contact health professionals and hospitals via email, however interestingly, they noted that the feature was not used often [21]. Thus, this area could be further explored.

In an eHealth program, it can be of interest to track participant progression throughout the program. For example, prehabilitation programs can determine whether participants are in a suitable position to safely have surgery while management platforms can track symptoms and disease progression. To ascertain participant progression in eHealth programs, a discussion board or a feedback form after every few modules could be useful. Another useful suggestion was for upper body exercises for participants unable to complete lower extremity exercises due to pain. While the suggestions offered are helpful, the expansion of HIPPER to include these features would have to be carefully evaluated to ensure the program remains at an appropriate length and does not become convoluted. The level of difficulty related to implementing these features would also have to be considered. These are important considerations when integrating user feedback during the development of eHealth programs and ensure that programs are clear and concise whilst delivering their goals effectively.

This qualitative study highlights the many benefits of prehabilitation education including participants feeling prepared for surgery and recovery. THR patients credit HIPPER for helping them manage their expectations and their successful post-surgical outcomes. A specific benefit of preoperative education is actively engaging patients with their recovery [44], and HIPPER included a module called ‘after surgery’ which contains helpful information for post-surgery expectations and management. However, interestingly, participants mentioned not revisiting the modules post-surgery. Instead, they mentioned that the current standard of care of pamphlets, exercise books and physiotherapy met all their needs post-surgery. Overall, by incorporating participant feedback, eHealth tools delivering educational prehabilitation such as HIPPER have the potential to be helpful, applicable, and accessible to patients, their caregivers and healthcare providers throughout BC and other parts of Canada. Individuals undergoing THR and other elective surgeries have diverse needs and surgical experiences, and eHealth tools may offer a solution to these challenges. The results of this study demonstrate the usability of eHealth by older adults and can also inform the development of other eHealth programs as well as the design of trials to test and implement such programs.

### Strengths and limitations

This study has some limitations. It was conducted during the peak of COVID-19 resulting in a fluctuating number of patients on the THR surgical waitlist. Sometimes participants would be scheduled for surgery without our team’s knowledge and as a result, some interviews were conducted months after their surgery. Thus, those participants may have forgotten certain aspects of the program, limiting the amount of information we could collect. However, some participants reviewed the modules prior to the interview to refresh their memory. The sample is not completely representative of the hip replacement population as we only included individuals at least 50 years old, limiting the transferability of our findings to younger individuals. However, this captures 80% of people with hip replacement. Not all HIPPER users agreed to participate in the interview, so our results may not represent the totality of opinions. Nevertheless, a majority of the HIPPER group (69%) agreed to an interview. Furthermore, while the intervention group was not supposed to attend in person education sessions, which form the current standard of care for THR preparation in BC, five participants did so anyway. This presents the notion that attending online and in person sessions could be complimentary. However, this number was only 19% of HIPPER users, and does impede the findings of the usability of HIPPER as an eHealth program.

## Conclusion

Prehabilitation offers a multitude of benefits by providing realistic expectations for patients and preparing them to have the best possible outcomes. Delivering a prehabilitation program through an eHealth platform improves accessibility, potential for cost-effectiveness and provides flexibility. Our results support the simple usability of eHealth programs such as HIPPER, especially amongst older adults. We demonstrated that eHealth programs that incorporate stakeholder perspectives can deliver effective educational prehabilitation programs for THR surgery and recovery. We found that an eHealth program enhanced participants’ confidence, reassured them, and prepared them for surgery and recovery. Features such as past patient interviews, equipment information, and practical resources were highly appreciated. This information can help health care providers understand what patients find useful to prepare for THR and inform the development of further eHealth tools. These encouraging results favor further development and implementation of HIPPER and similar eHealth programs. Future work should focus on conducting large scale studies to explore what motivates patients to complete these programs and facilitators to their successful implementation in existing healthcare systems.

## References

[1] Public Health Agency of Canada. Osteoarthritis in Canada [Internet]; 2020 [cited 2023 Oct 4]. Available from: https://www.canada.ca/en/public-health/services/publications/diseases-conditions/osteoarthritis.html

[2] Felson DT, Lawrence RC, Hochberg MC, McAlindon T, Dieppe PA, Minor MA. Osteoarthritis: new insights. Part 2: treatment approaches. Ann Intern Med. 2000;133(9):726–37. 11074906 10.7326/0003-4819-133-9-200011070-00015

[3] Laupacis A, Bourne R, Rorabeck C, Feeny D, Wong C, Tugwell P, et al. The effect of elective total hip replacement on health-related quality of life. J Bone Joint Surg Am. 1993;75(11):1619–26. doi: 10.2106/00004623-199311000-00006 8245054

[4] Canadian Institute for Health Information. Hospital stays in Canada [Internet]; 2022 [cited 2023 Oct 4]. Available from: https://www.cihi.ca/en/hospital-stays-in-canada

[5] Canadian Institute for Health Information. CJRR annual report: hip and knee replacements in Canada, 2020–2021 [Internet]; 2022 [cited 2023 Oct 4]. Available from: https://www.cihi.ca/en/cjrr-annual-report-hip-and-knee-replacements-in-canada-2020-2021

[6] Canadian Institute for Health Information. COVID-19’s impact on hospital services [Internet]; 2021 [cited 2023 Oct 4]. Available from: https://www.cihi.ca/en/covid-19-resources/impact-of-covid-19-on-canadas-health-care-systems/hospital-services

[7] Banugo P, Amoako D. Prehabilitation. BJA Educ. 2017;17(12):401–5.

[8] Ditmyer MM, Topp R, Pifer M. Prehabilitation in preparation for orthopaedic surgery. Orthop Nurs. 2002;21(5):43–51; quiz 52–4. doi: 10.1097/00006416-200209000-00008 12432699

[9] Widmer P, Oesch P, Bachmann S. Effect of prehabilitation in form of exercise and/or education in patients undergoing total hip arthroplasty on postoperative outcomes-a systematic review. Medicina (Kaunas). 2022;58(6):742. doi: 10.3390/medicina58060742 35744005 PMC9228426

[10] Santa Mina D, Scheede-Bergdahl C, Gillis C, Carli F. Optimization of surgical outcomes with prehabilitation. Appl Physiol Nutr Metab. 2015;40(9):966–9. doi: 10.1139/apnm-2015-0084 26300015

[11] Carli F, Scheede-Bergdahl C. Prehabilitation to enhance perioperative care. Anesthesiol Clin. 2015;33(1):17–33. doi: 10.1016/j.anclin.2014.11.002 25701926

[12] Cabilan CJ, Hines S, Munday J. The impact of prehabilitation on postoperative functional status, healthcare utilization, pain, and quality of life: a systematic review. Orthop Nurs. 2016;35(4):224–37. doi: 10.1097/NOR.0000000000000264 27441877

[13] Durrand J, Singh S, Danjoux G. Prehabilitation. Clin Med. 2019;19(6):458–64. 10.7861/clinmed.2019-0257 PMC6899232 31732585

[14] van Germert-Pijnen L (JEWC), Kip H, Kelders SM, Sanderman R. Introducing eHealth. In: eHealth research, theory and development. Routledge; 2018.

[15] Oh H, Rizo C, Enkin M, Jadad A. What is eHealth: a systematic review of published definitions. J Med Internet Res. 2005;7(1):e110. doi: 10.2196/jmir.7.1.e110 PMC1550636 15829471

[16] Waller A, Forshaw K, Carey M, Robinson S, Kerridge R, Proietto A. Optimizing patient preparation and surgical experience using eHealth technology. JMIR Med Inform. 2015;3(3):e4286. doi: 10.2196/medinform.4286 PMC4705017 26330206

[17] Koutras C, Bitsaki M, Koutras G, Nikolaou C, Heep H. Socioeconomic impact of e-Health services in major joint replacement: a scoping review. Technol Health Care. 2015;23(6):809–17. 26409523 10.3233/THC-151036

[18] Mohammadi S, Miller WC, Wu J, Pawliuk C, Robillard JM. Effectiveness of eHealth tools for hip and knee arthroplasty: a systematic review. Front Rehabil Sci [Internet]. 2021 [cited 2023 Oct 5];2:696019. Available from: https://www.frontiersin.org/articles/10.3389/fresc.2021.696019 36188859 10.3389/fresc.2021.696019 PMC9397702

[19] Gorton J. Introducing virtual prehabilitation to a UK elective total hip replacement service to improve patient outcomes: an overview of the literature and proposed implementation plan. J Rehabil. 2022 [cited 2023 Oct 5]. Available from: https://journals.sagepub.com/doi/10.1177/22104917221075830

[20] Doiron-Cadrin P, Kairy D, Vendittoli P-A, Lowry V, Poitras S, Desmeules F. Feasibility and preliminary effects of a tele-prehabilitation program and an in-person prehablitation program compared to usual care for total hip or knee arthroplasty candidates: a pilot randomized controlled trial. Disabil Rehabil. 2020;42(7):989–98. doi: 10.1080/09638288.2018.1515992 30638076

[21] Saunders R, Seaman K, Emery L, Bulsara M, Ashford C, McDowall J, et al. Comparing an eHealth program (my hip journey) with standard care for total hip arthroplasty: randomized controlled trial. JMIR Rehabil Assist Technol. 2021;8(1):e22944. doi: 10.2196/22944 33656449 PMC8082385

[22] Reid H, Mohammadi S, Watson W, Robillard JM, Crocker M, Westby MD, et al. Patient and caregiver perspectives on an eHealth tool: a qualitative investigation of preferred formats, features and characteristics of a presurgical ehealth education module. Rehabil Process Outcome. 2021 [cited 2023 Oct 5];10:11795727211010501. Available from: https://journals.sagepub.com/doi/10.1177/11795727211010501 34497456 10.1177/11795727211010501 PMC8282173

[23] Doyle L, McCabe C, Keogh B, Brady A, McCann M. An overview of the qualitative descriptive design within nursing research. Journal of Research in Nursing. 2019;25(5): 443–455. doi: 10.1177/1744987119880234 34394658 PMC7932381

[24] Miller WC, Mohammadi S, Watson W, Crocker M, Westby M. Correction: the Hip Instructional Prehabilitation Program for Enhanced Recovery (HIPPER) as an eHealth approach to presurgical hip replacement education: protocol for a randomized controlled trial. JMIR Res Protoc. 2022;11(7):e39745. doi: 10.2196/39745 35834805 PMC9335173

[25] O’Brien BC, Harris IB, Beckman TJ, Reed DA, Cook DA. Standards for reporting qualitative research: a synthesis of recommendations. Acad Med. 2014;89(9):1245–51. doi: 10.1097/ACM.0000000000000388 24979285

[26] Hsieh H-F, Shannon SE. Three approaches to qualitative content analysis. Qual Health Res. 2005 [cited 2023 Oct 5];15(9):1277–88. doi: 10.1177/1049732305276687 16204405

[27] Salkind NJ. Encyclopedia of research design. Los Angeles (CA): SAGE; 2010. xxxi+1719 p.

[28] Dodgson JE. Reflexivity in qualitative research. J Hum Lact. 2019 [cited 2023 Oct 5];35(2):220–2. Available from: https://journals.sagepub.com/doi/10.1177/0890334419830990 30849272 10.1177/0890334419830990

[29] Fouquet SD, Miranda AT. Asking the right questions-human factors considerations for telemedicine design. Curr Allergy Asthma Rep. 2020;20(11):66. doi: 10.1007/s11882-020-00965-x 32862299 PMC7456356

[30] Rangachari P. A Holistic framework of strategies and best practices for telehealth service design and implementation. In: Pfannstiel MA, Brehmer N, Rasche C, editors. Service design practices for healthcare innovation: paradigms, principles, prospects [Internet]. Cham: Springer International Publishing; 2022 [cited 2023 Oct 5]. p. 315–35. Available from: doi: 10.1007/978-3-030-87273-1_16

[31] Hiltz SR. Impacts of college-level courses via asynchronous learning networks: some preliminary results. Online Learn [Internet]. 1997 [cited 2023 Oct 5];1(2). Available from: https://olj.onlinelearningconsortium.org/index.php/olj/article/view/1934

[32] Dunlosky J, Rawson KA, Marsh EJ, Nathan MJ, Willingham DT. Improving students’ learning with effective learning techniques: promising directions from cognitive and educational psychology. Psychol Sci Public Interest. 2013 [cited 2023 Oct 5];14(1):4–58. Available from: https://journals.sagepub.com/doi/10.1177/1529100612453266 26173288 10.1177/1529100612453266

[33] Lindsey RV, Shroyer JD, Pashler H, Mozer MC. Improving students’ long-term knowledge retention through personalized review. Psychol Sci. 2014 [cited 2023 Oct 5];25(3):639–47. Available from: https://journals.sagepub.com/doi/10.1177/0956797613504302 24444515 10.1177/0956797613504302

[34] Diep NA, Cocquyt C, Zhu C, Vanwing T, de Greef M. Effects of core self-evaluation and online interaction quality on adults’ learning performance and bonding and bridging social capital. Internet High Educ. 2017;34(1):41–55.

[35] Hodges C. Self-efficacy, motivational email, and achievement in an asynchronous math course. J Comput Math Sci Teach. 2008;27(3):265–85.

[36] Kim M, Ketenci T. Learner participation profiles in an asynchronous online collaboration context. Internet High Educ. 2019;41(1):62–76.

[37] Pilotti M, Anderson S, Hardy P, Murphy P, Vincent P. Factors related to cognitive, emotional, and behavioral engagement in the online asynchronous classroom; 2017.

[38] Xie K, Durrington V, Yen L. Relationship between students’ motivation and their participation in asynchronous online discussions. Online Learn. 2011;7:17–29.

[39] Brewer S, Klein J. Type of positive interdependence and affiliation motive in an asynchronous, collaborative learning environment. Educ Technol Res Dev. 2006;54(4):331–54.

[40] Lewis C, Menn L. Access tool? Accelerating treadmill? Technology and the aging population. In: Stephanidis C, editor. Universal access in human-computer interaction addressing diversity. Berlin, Heidelberg: Springer; 2009. p. 263–8. (Lecture Notes in Computer Science).

[41] Mace RA, Mattos MK, Vranceanu AM. Older adults can use technology: why healthcare professionals must overcome ageism in digital health. Transl Behav Med. 2022:ibac070. 36073770 10.1093/tbm/ibac070 PMC9494377

[42] Statistics Canada. Internet use by location of use, age group, household income and geography, inactive [Internet]; 2011 [cited 2023 Oct 5]. Available from: https://www150.statcan.gc.ca/t1/tbl1/en/tv.action?pid=2710001701

[43] Sousa V, Lopez K. Towards usable e-health. Appl Clin Inform. 2017;8(2):470–90. 28487932 10.4338/ACI-2016-10-R-0170 PMC6241759

[44] Poland F, Spalding N, Gregory S, McCulloch J, Sargen K, Vicary P. Developing patient education to enhance recovery after colorectal surgery through action research: a qualitative study. BMJ Open. 2017;7(6):e013498. doi: 10.1136/bmjopen-2016-013498 28667197 PMC5577868

